# Gut microbial metabolite deoxycholic acid facilitates Th17 differentiation through modulating cholesterol biosynthesis and participates in high-fat diet-associated colonic inflammation

**DOI:** 10.1186/s13578-023-01109-0

**Published:** 2023-10-03

**Authors:** Dan Li, Jiefei Zhou, Lingyu Wang, Zizhen Gong, Huijuan Le, Ye Huang, Congfeng Xu, Chunyan Tian, Wei Cai, Jin Wu

**Affiliations:** 1 Department of Pediatric Surgery, Xinhua Hospital, School of Medicine, Shanghai Jiaotong University, Shanghai, China; 2 Shanghai Institute for Pediatric Research, School of Medicine, Shanghai Jiaotong University, Shanghai, China; 3 Shanghai Key Laboratory of Pediatric Gastroenterology and Nutrition, Shanghai, China; 4 Department of Cardiology, Shanghai Jiao Tong University Affiliated Sixth People’s Hospital, Shanghai, China; 5 State Key Laboratory of Proteomics, Beijing Proteome Research Center, National Center for Protein Sciences (Beijing), Beijing Institute of Lifeomics, Beijing, China; 6 Research Unit of Proteomics-Driven Cancer Precision Medicine, Chinese Academy of Medical Sciences, Beijing, China

**Keywords:** Colonic inflammation, High fat diet, Bile acid, Th17 differentiation

## Abstract

**Background:**

High-fat diet (HFD) is closely associated with the increased prevalence of inflammatory bowel disease (IBD). Excessive gut microbial metabolite deoxycholic acid (DCA) caused by HFD plays significant roles in eliciting intestinal inflammation, however, the mechanism underlining the induction of inflammatory response by DCA has not been fully elucidated. The purpose of this study was to investigate the role of DCA in the triggering of inflammation via affecting CD4^+^ T cell differentiation.

**Results:**

Murine CD4^+^T cells were cultured under Th1, Th2 or Th17-polarizing conditions treated with or without different dosage of DCA, and flowcytometry was conducted to detect the effect of DCA on CD4^+^ T cell differentiation. Alteration of gene expression in CD4^+^ T cells upon DCA treatment was determined by RNA-sequencing and qRT-PCR. Bioinformatic analysis, cholesterol metabolic profiling, ChIP assay and immuno-fluorescent staining were further applied to explore the DCA-regulated pathway that involved in CD4^+^T cell differentiation. The results showed that DCA could dose-dependently promote the differentiation of CD4^+^ T cell into Th17 linage with pathogenic signature. Mechanistically, DCA stimulated the expression of cholesterol biosynthetic enzymes CYP51 and led to the increased generation of endogenous RORγt agonists, including zymosterol and desmosterol, therefore facilitating Th17 differentiation. Up-regulation of CYP51 by DCA was largely mediated via targeting transcription factor SREBP2 and at least partially through bile acid receptor TGR5. In addition, DCA-supplemented diet significantly increased intestinal Th17 cell infiltration and exacerbated TNBS-induced colitis. Administration of cholestyramine to eliminate fecal bile acid obviously alleviated colonic inflammation accompanied by decreased Th17 cells in HFD-fed mice.

**Conclusions:**

Our data establish a link between DCA-induced cholesterol biosynthesis in immune cells and gut inflammation. Modulation of bile acid level or targeting cholesterol metabolic pathway may be potential therapeutic measurements for HFD-related colitis.

**Supplementary Information:**

The online version contains supplementary material available at 10.1186/s13578-023-01109-0.

## Background

Westernized high-fat diet (HFD) is deemed as an important environmental factor contributing to the rapid increase in the incidence of inflammatory bowel disease (IBD) [1–6]. High-fat diet intake could induce systemic low-grade inflammation, and colon is considered to be the first organ suffering from inflammation due to HFD [7]. Increasing evidence indicates that the alteration of gut microbiota and consequently elevating of microbial metabolites deoxycholic acid (DCA) induced by HFD play significant roles in inducing intestinal inflammation and colorectal malignancies [8–11]. DCA is the major bile acid in human feces, moreover, fecal concentration of DCA can increase nearly tenfold under HFD conditions, and long-term exposure to the high level colonic DCA in mice could evoke obvious colonic inflammation that resembles human IBD [12–14]. However, the mechanisms underlining the induction of inflammatory response by DCA have not been fully understood.

Since innate immune system and adaptive immune system are coordinately responsible for the orchestration of gut homeostasis, the occurrence of intestinal inflammation commonly attributes to the imbalance of intestinal mucosal immune response. High level DCA correlates with intestinal inflammation, suggesting its regulatory effects on gut mucosal immune cells. Our previous studies have proved that high level DCA could act as damage-associated molecular pattern (DAMP) to trigger NLRP3 inflammasome activation in macrophages, meanwhile, excessive DCA could also trans-activate toll-like receptor 2 (TLR2) and downstream signaling, promoting M1 macrophage polarization as well as pro-inflammatory cytokines production and therefore participating in the HFD-related colonic inflammation [15, 16].

Intestinal inflammation also involves adaptive immune compartments, especially CD4^+^T-helper (Th) cells. Upon activation, CD4^+^T cells can further differentiate into various subsets, mainly including Th1, Th2 and Th17 cells, which produce distinct cytokines and are proved to be implicated in the development and deterioration of intestinal inflammation in IBD patients and disease mouse models [17]. Th1 cells are generally believed to participate in the pathogenesis of Crohn's disease (CD) by secreting IFN-γ and TNF-α, whereas ulcerative colitis (UC) is mainly mediated by Th2 cells that produce IL-4, IL-5 and IL-13 [18]. In addition, accumulating studies have emphasized the crucial role of Th17 cells in diverse inflammatory diseases, including IBD. Clinical studies find increased Th17 cells and related cytokines (such as IL-17, IL-21) in the serum and inflamed colonic tissues of IBD patients (UC and CD), and Th17 cell proportions as well as related cytokine levels are correlated well with disease activity [19–21]. Given the importance of CD4^+^T cells in intestinal inflammation, we sought to investigate whether high level DCA-induced colonic inflammation also involves its effects on CD4^+^T cells differentiation.

Here our study demonstrates that DCA can dose-dependently promote Th17 cell differentiation and contribute to the colonic inflammation and tissue injury. The effect of DCA is mainly achieved through enhancing cholesterol biosynthesis in CD4^+^T cells by modulating the expression of cholesterol biosynthetic enzymes, especially CYP51, thereby resulting in the increased production of endogenous activating ligands of ROR γt. The up-regulation of CYP51 level by DCA is mediated via targeting transcription factor SREBP2 and at least partially through bile acid receptor TGR5.

## Results

### DCA enhances Th17 cell differentiation

**Fig. 1:**
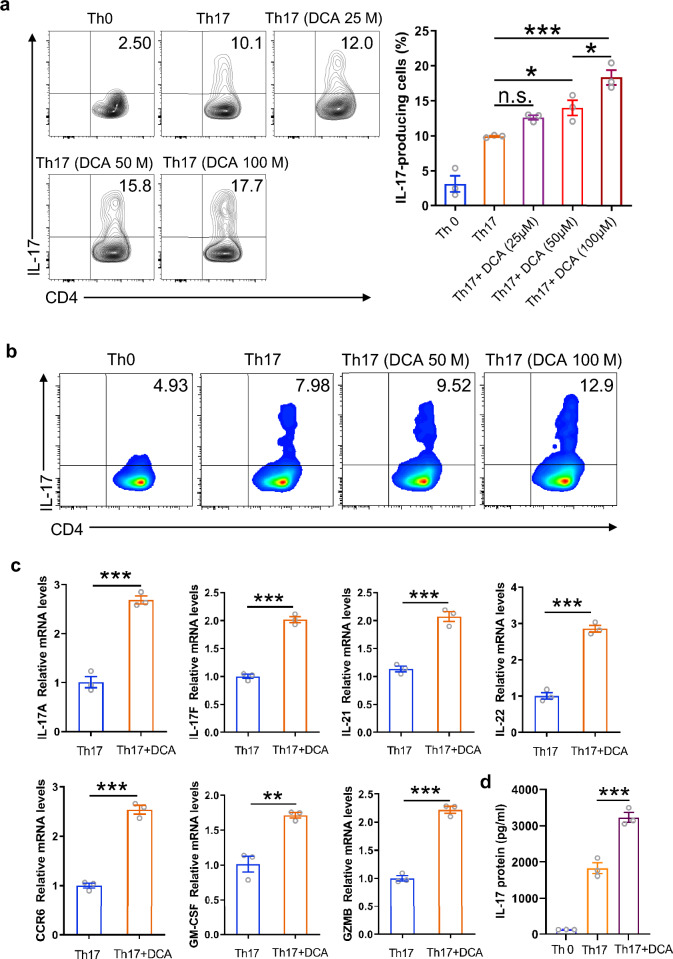
DCA promotes Th17 cell differentiation. **a** Splenocytes were differentiated under Th0 or Th17-polarizing conditions for 72 h in the absence or presence of different dosage of DCA, then the cells were re-stimulated with PMA/ionomycin and analyzed for the percentages of IL-17-producing CD4^+^ T cells by flow cytometry (n = 3). **b** Naïve CD4^+^ T cells were differentiated under Th0 or Th17-polarizing conditions in the absence or presence of DCA. Cells were then re-stimulated with PMA/ionomycin and the percentages of IL-17-producing CD4^+^ T cells were determined. **c** Naïve CD4^+^ T cells were differentiated under Th17-polarizing conditions for 72 h in the absence or presence of DCA (100 µM), then the cells were re-stimulated with PMA/ionomycin for 5 h and mRNA expression levels of indicated genes was detected by real-time PCR (n = 3). **d** Naïve CD4^+^ T cells were differentiated under Th0 or Th17-polarizing conditions for 72 h in the absence or presence of DCA (100 µM). Cells were then re-stimulated with PMA/ionomycin for 12 h and the supernatants were analyzed for IL-17 by ELISA (n = 3). *p < 0.05; **p < 0.01; ***p < 0.001. n.s.: no statistically significant difference (*p* > 0.05). One-way ANOVA with Tukey's multiple comparisons tests (**a** right panel, **d**) or two-tailed Student’s *t*-tests were used (**c**). Data are expressed as mean ± SEM from at least three independent experiments or representative data (**a**: left panel, **b**)

**Fig. 2:**
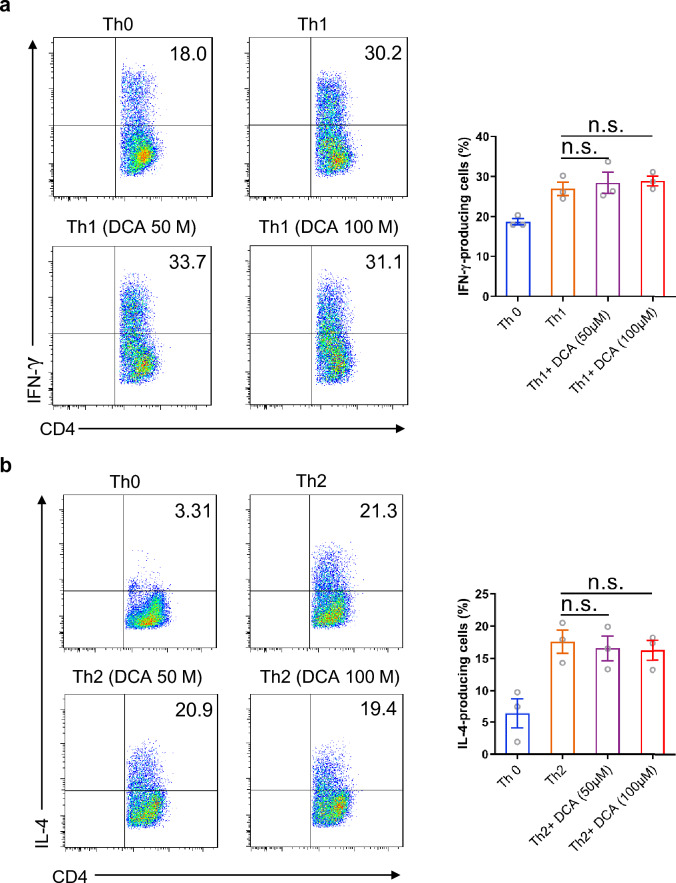
The effect of DCA on Th1 and Th2 cell differentiation. **a** Splenocytes were differentiated under Th0 or Th1-polarizing conditions for 72 h in the absence or presence of different dosage of DCA, then the cells were re-stimulated with PMA/ionomycin and analyzed for the percentages of IFN γ-producing CD4^+^ T cells by flow cytometry (n = 3). **b** Splenocytes were differentiated under Th0 or Th2-polarizing conditions for 72 h in the absence or presence of different dosage of DCA, then the cells were re-stimulated with PMA/ionomycin and analyzed for the percentages of IL-4-producing CD4^+^ T cells by flow cytometry (n = 3). n.s.: no statistically significant difference (*p* > 0.05). One-way ANOVA with Tukey's multiple comparisons tests were used (**a** and **b** right panel). Data are expressed as mean ± SEM from at least three independent experiments or representative data (**a** and **b** left panel)

Considering the importance of T cells in intestinal inflammation, we sought to explore whether DCA was able to modulate CD4^+^T cell differentiation. Murine splenocytes were primed for 72 h under Th0 or Th1, Th2, Th17-polarizing conditions in the absence or presence of different dosage of DCA, then the cells were re-stimulated with PMA/ionomycin and analyzed for the percentages of IFN γ-producing, IL-4-producing or IL-17-producing CD4^+^ T cells respectively by flow cytometry. Of note, the frequency of IL-17-producing cells was significantly increased upon DCA stimulation in a dose-dependent manner (Fig. 1a), meanwhile, DCA exhibited similar effects on CD4^+^T cells purified from murine spleen and lymph nodes (Fig. 1b). Consistently, transcript levels of the Th17 signature cytokines, including IL-17A, IL-17F, IL-21 and IL-22, were strongly elevated in DCA-treated cells (Fig. 1c), and the elevation of IL-17 protein level was confirmed by ELISA (Fig. 1d). In addition, high level DCA also significantly up-regulated molecules related to the pathogenicity of Th17 cells, such as CCR6, a chemokine receptor crucial for pro-inflammatory function of Th17 in autoimmune disease involving EAE and arthritis, and GM-CSF as well as GZMB, the critical components of pathogenic Th17 signature [22–24] (Fig. 1c). In contrast, Th1 or Th2 differentiation was not obviously affected by DCA stimulation, as evidenced by the comparable percentages of IFN γ-producing cells/IL-4-producing cells with or without DCA treatment under Th1/Th2 inducing conditions (Fig. 2a, b). Taken together, these data indicate that high level DCA could promote the differentiation of CD4^+^T cell to the pathogenic Th17 subset.

### Cholesterol biosynthetic enzyme CYP51 is up-regulated upon DCA stimulation and involved in DCA-promoted Th17 cell differentiation

**Fig. 3:**
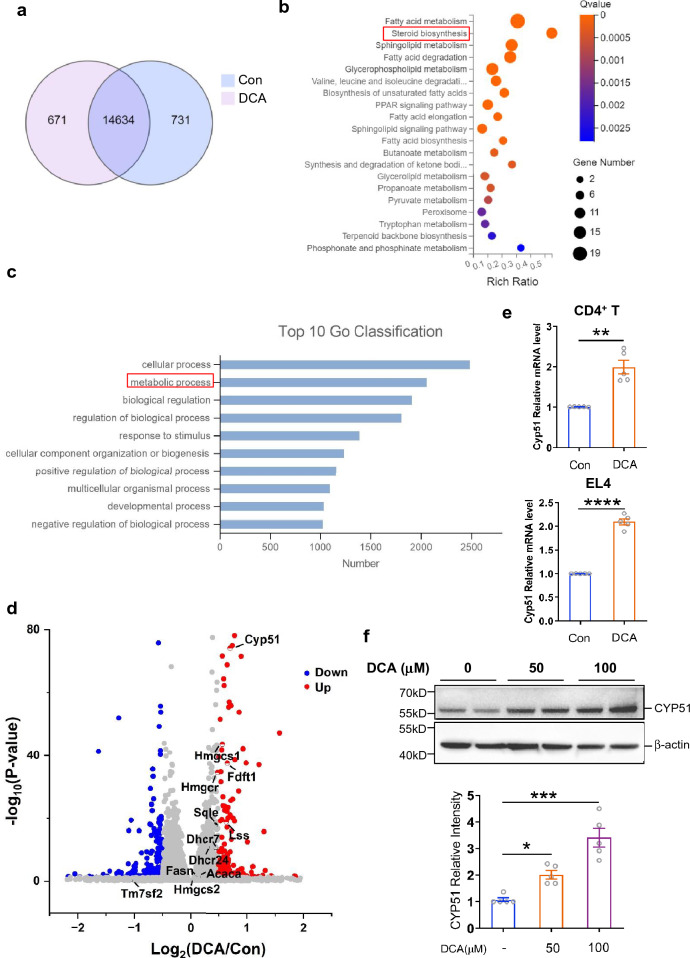
Cholesterol biosynthetic enzyme CYP51 is up-regulated upon DCA stimulation. **a** Venn diagram showing the overlap of genes in untreated and DCA-treated CD4^+^T cells (n = 3). KEGG pathways **b** and GO terms **c** enrichment analysis for DEGs (differentially expressed genes) induced by DCA. **d** Volcano plot of differentially expressed genes. Red and green dots respectively represent up-regulated and down-regulated DEGs upon DCA treatment. Grey dots indicate the unchanged genes. The cholesterol biosynthesis related genes were labeled. **e** Murine CD4^+^T cells or EL4 cells were treated with or without DCA (100µM) for 4h. The mRNA expression of CYP51 was determined by real-time PCR (n = 5). **f** EL4 cells were treated with different dosages of DCA for 24h. The protein expression level of CYP51 was determined by western blot (n = 5). *p < 0.05; **p < 0.01; ***p < 0.001; ****p < 0.0001. Two-tailed Student’s *t*-tests (**e**) or one-way ANOVA with Tukey's multiple comparisons test (**f** bottom panel) was used. Data are expressed as mean ± SEM from at least three independent experiments or representative data (**f** upper panel)

**Fig. 4:**
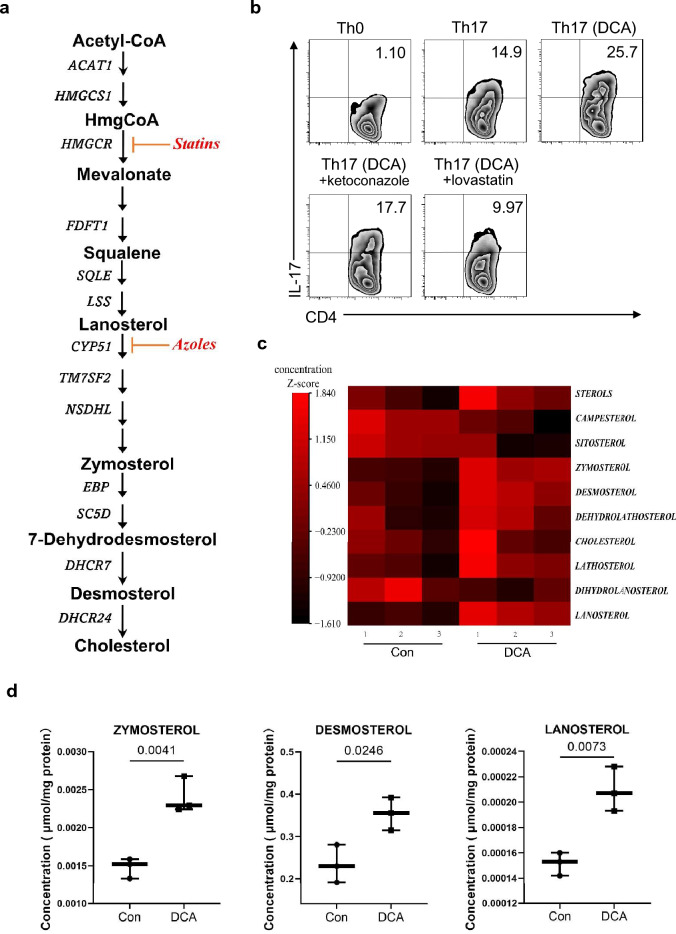
DCA increases the generation of endogenous RORγt agonists. **a** Schematic view of cholesterol synthetic pathways. Inhibitors used in **b** was shown in red. **b** CD4^+^ T cells were differentiated under Th0 or Th17-polarizing conditions in the absence or presence of DCA (100 µM). CYP51 inhibitor ketoconazole (1 µM) or cholesterol biosynthesis inhibitor lovastatin (10 µM) was added 30 min ahead of DCA treatment. The percentages of IL-17-producing CD4^+^ T cells were analyzed by flow cytometry. **c** Relative levels of cholesterol biosynthesis precursors in untreated and DCA-treated EL4 cells measured by LC–MS (n = 3). **d** Comparison of zymosterol, desosterol and lanosterol concentrations in untreated and DCA-treated EL4 cells (n = 3). Two-tailed Student’s *t-*tests were used. Data are expressed as mean ± SEM from at least three independent experiments or representative data (b)

To explore the possible mechanisms involved in the DCA-enhanced Th17 differentiation, primed murine CD4^+^T cells were stimulated with or without DCA and differential gene expression was determined by RNA-sequencing (Fig. 3a–d). Importantly, GO classification analysis together with KEGG pathways analysis showed that metabolic process related genes, especially those involved in steroid biosynthesis pathway, were significantly enriched (Fig. 3b, c). Heat map and volcano plot based on RNA-seq data showed that the expression of many cholesterol biosynthetic enzymes increased, especially CYP51 (lanosterol 14alpha -demethylase), which represented the most potently up-regulated molecule induced by DCA, whereas the expression of genes involved in cholesterol efflux, transport, esterification and oxidization was not apparently affected (Additional file 1: Fig. S1 and Fig. 3d). To validate the effect of DCA on CYP51 gene expression, real-time PCR was performed on control and DCA-treated CD4^+^T cells as well as EL4 cell line. In line with the RNA-seq data, mRNA expression level of CYP51 was indeed substantially up-regulated in response to DCA in both cells (Fig. 3e). Western-blot analysis also confirmed that DCA could increase CYP51 protein expression in a dose-dependent manner (Fig. 3f and Additional file 1: Fig. S2). Intriguingly, it has been reported that CYP51 is engaged in Th17 differentiation by facilitating generation of endogenous RORγt agonists during cholesterol biosynthesis process, and *Cyp51* deficiency can lead to reduced Th17 differentiation [25]. Here our results showed that CYP51 inhibitor could obviously hinder DCA-enhanced Th17 differentiation (Fig. 4a, b), therefore emphasizes the critical role of cholesterol biosynthetic enzyme CYP51 in the process of DCA-rendered Th17 differentiation.

Retinoic acid receptor-related orphan receptor γt (RORγt) functions as the key transcription factor for Th17 commitment and increasing evidence suggests the prominent effect of cholesterol metabolic pathway on Th17 differentiation, which attributes to the production of natural RORγt agonists [26–29]. As mentioned above, our data indicate that upon DCA stimulation, cholesterol biosynthesis pathway were activated and CYP51, the critical enzyme responsible for the generation of RORγt agonists, was potently up-regulated, subsequently we sought to confirm the role of cholesterol metabolism in DCA-enhanced Th17 differentiation and figure out whether DCA could increase the production of RORγt agonists. Therefore, lovastatin was applied to inhibit cholesterol biosynthesis and the result showed that cholesterol biosynthesis blockage could completely reverse the effect of DCA (Fig. 4a, b), meanwhile, DCA treatment dramatically increased the generation of zymosterol and desmosterol, two cholesterol biosynthetic intermediates regarded as the most effective sterols activating RORγt (Fig. 4c, d). In addition, the amount of weak RORγt agonist lanosterol was also strongly elevated (Fig. 4d). These findings suggest that DCA-enhanced Th17 cell differentiation may correlate with increased generation of endogenous RORγt agonists during cholesterol biosynthetic process.

### DCA increases CYP51 expression by targeting transcriptional factor SREBP2

**Fig. 5:**
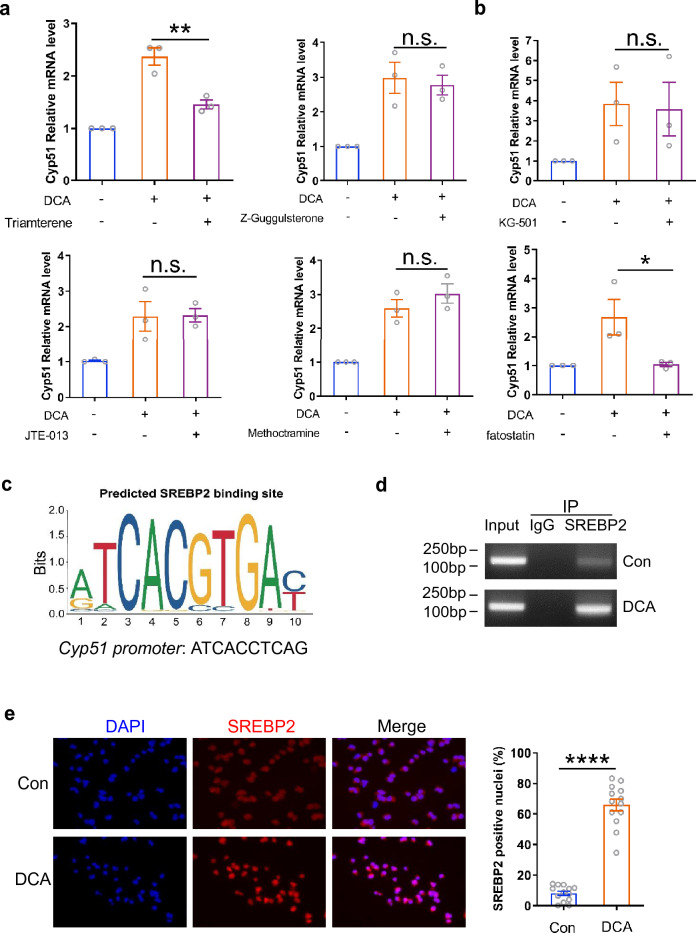
DCA increases CYP51 expression by targeting transcriptional factor SREBP2. **a** EL4 cells were stimulated with DCA (100 µM) in the presence or absence of Triamterene (10 μM, TGR5 inhibitor), Z-Guggulsterone (20 μM, FXR inhibitor), JTE-013 (10 μM, S1PR2 inhibitor) or methoctramine (5 µM, M2-mAchR inhibitor). The mRNA expression levels of CYP51 were determined by real-time PCR (n = 3). **b** EL4 cells were stimulated with DCA (100 µM) in the presence or absence of KG-501 (10 µM) or fatostatin (10 µM). The mRNA expression levels of CYP51 were determined by real-time PCR (n = 3). **c** Predicted SREBP2 binding site on *Cyp51* promoter. Colorful letters were the motif of SREBF2 binding site cited from JASPAR. **d** EL4 cells were stimulated with or without DCA (100 µM) for 24 h and then ChIP assay was performed. Anti-SREBP2 antibody or isotype-matched IgG control antibody were used. PCR was applied to quantify the precipitated DNA with primers flanking the SREBP2 binding region of the CYP51 promoter. **e** Representative SREBP2 (red) and DAPI (blue) immunofluorescence staining of EL4 cells untreated or treated with DCA (n = 3). Data are pooled from three independent experiments. Each dot represents one microscopic high power field (HPF); four to five HPFs were scored per experiment and totally at least 300 cells were evaluated. *p < 0.05; **p < 0.01; ****p < 0.0001. n.s.: no statistically significant difference (*p* > 0.05). One-way ANOVA with Tukey's multiple comparisons tests (a, b) or two-tailed Student’s *t*-test with Welch's correction (e: right panel) was used. Data are expressed as mean ± SEM from at least three independent experiments or representative data (d, e: left panel)

To further figure out the molecular mechanism underlying DCA-regulated CYP51 expression, bile acid membrane and nuclear receptors including TGR5, Sphingosine-1-phosphate receptor 2 (S1PR2), M2 muscarinic acetylcholine receptor (M2-mAchR) and farnesoid X-receptor (FXR) were inhibited respectively [30, 31]. We found that selective inhibition of TGR5 with triamterene significantly reduced the expression of CYP51 enhanced by DCA, while blockage of other bile acid receptors mentioned above with corresponding specific inhibitors had no obvious effect (Fig. 5a), indicating that DCA promotes CYP51 expression at least partially through TGR5. In addition, cAMP responsive element binding protein (CREB) and sterol-regulatory element binding protein (SREBP) represent two kinds of major transcription factors responsible for the tight control of CYP51 mRNA transcription [32], to better understand the regulatory mechanism of CYP51 gene transcription by DCA, specific CREB or SREBP inhibitor was added to the culture medium 30 min before DCA treatment. Notably, the results exhibited that inhibition of SREBP but not CREB completely eliminated the regulatory effect of DCA on CYP51 mRNA expression (Fig. 5b). Since SREBP2 plays a predominant role in the control of cholesterol biosynthesis, and bioinformatics analysis revealed important SREBP binding sequences (ATCACCTCAG) at promoter region of *Cyp51* (Fig. 5c), chromatin immunoprecipitation (ChIP) assay was then performed using a SREBP2-specific antibody. Data showed that SREBP2 antibody pulled down much more predicted binding region sequences on *Cyp51* promoter in DCA-treated cells than untreated control (Fig. 5d). Immunofluorescent staining further confirmed that DCA treatment could induce obvious translocation of SREBP2 into nucleus, whereas SREBP2 primarily remained in the cytoplasm of control cells (Fig. 5e). These data highly imply that DCA regulates CYP51 gene transcription mainly by targeting SREBP2.

### Cholestyramine treatment alleviates HFD-associated colonic inflammation

**Fig. 6:**
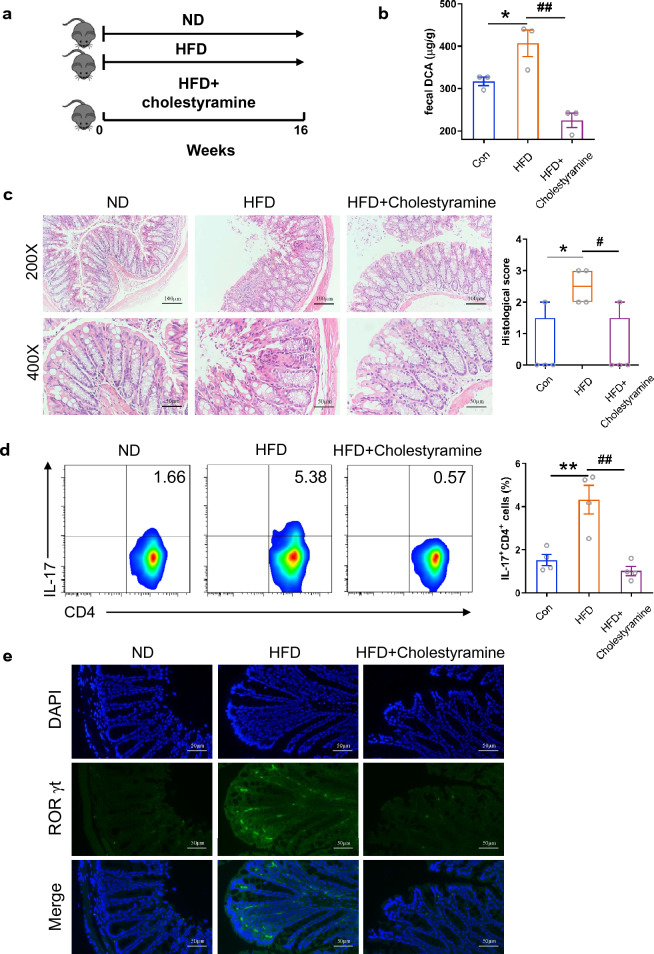
Cholestyramine treatment alleviates HFD-associated colonic inflammation. **a** Animal treatment procedure (ND, n = 5; HFD, n = 6; HFD + VCM, n = 7). **b** Fecal DCA concentrations (n = 3). **c** Representative HE staining and histological score of colon sections from ND, HFD and HFD plus cholestyramine treated mice (n = 4). Scale bar, 100 µm (×200) and 50 µm (×400). **d** The percentage of IL-17-producing cells from mesenteric lymph nodes of ND, HFD and HFD plus cholestyramine treated mice (n = 4). **e** Representative RORγt (green) and DAPI (blue) immunofluorescence staining of colon tissues from ND, HFD and HFD plus cholestyramine treated mice (Scale bar, 50 µm). *p < 0.05; **p < 0.01 compared to the normal diet control mice. ^#^*p* < 0.05; ^##^p < 0.01 compared to the HFD plus cholestyramine treated mice. One-way ANOVA with Newman-Keuls multiple comparisons test (b) or Tukey's multiple comparisons tests (c, d) were used. Data are expressed as mean ± SEM from at least three independent experiments or representative data (**c** and **d**: left panel, **e**)

It has been reported that HFD could aggravate colitis by selectively promoting Th17 differentiation [33], however, the exact triggers remain elusive. To ascertain the role of bile acid in the HFD-associated colonic inflammation, mice were fed with HFD for 4 months and some of which were orally administrated with cholestyramine, a bile acid sequestrant, to promote fecal bile acid excretion (Fig. 6a). Compared to the normal chow diet, long term HFD feedings induced obvious increment of fecal DCA and inflammatory cell infiltration as well as tissue damage in the colon (Fig. 6b, c). In addition, HFD diet also significantly increased the percentage of Th17 cells in mesenteric lymph nodes, meanwhile, immunofluorescence staining for RORγt, the Th17 lineage-specific transcription factor, showed increment of RORγt^+^ Th17 cell accumulation in colon tissue of HFD-fed mice (Fig. 6d, e). Notably, cholestyramine treatment efficiently decreased DCA level and ameliorated intestinal damage accompanied by profound inhibition of Th17 cell generation (Fig. 6b–e). Since dietary fat has been observed to mainly increase the level of fecal secondary bile acids, especially DCA [12], which may encounter immune cells in the colonic lamina propria when undergo reabsorption through passive diffusion, and our in vitro data proved that high level DCA could promote pathogenic Th17 differentiation, these findings suggest that fecal bile acid, particularly DCA, may be involved in the in vivo pro-inflammatory Th17 cells formation and colonic inflammation under HFD settings.

### DCA administration enhances Th17 immune response and exacerbates TNBS-induced colitis

**Fig. 7:**
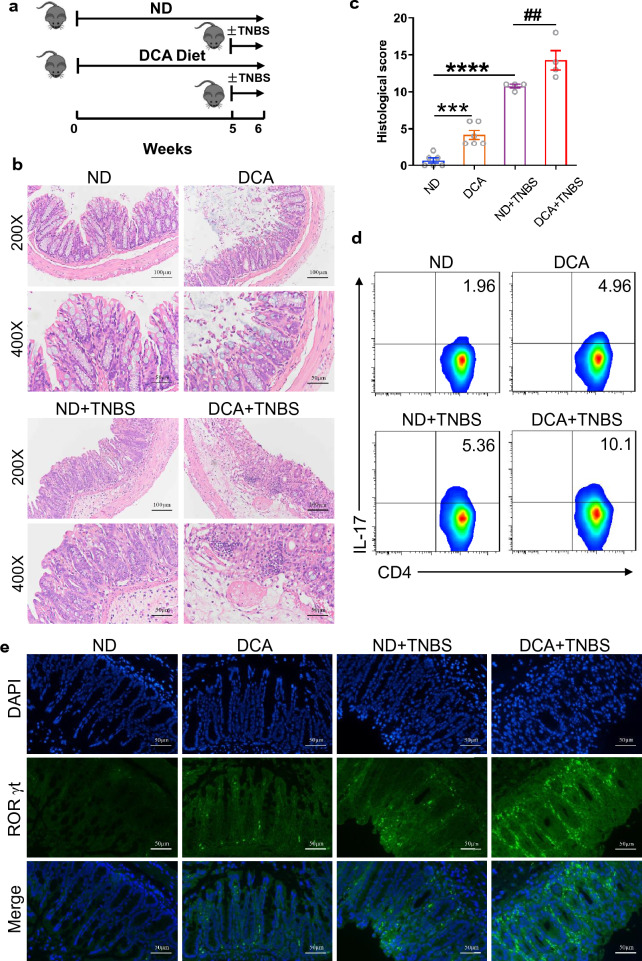
DCA administration enhances Th17 immune response and exacerbates TNBS-induced colitis. **a** Animal treatment procedure (n = 7 per group). **b** Representative HE staining and **c** histological score of colon sections from ND, DCA, ND + TNBS and DCA + TNBS treated mice (n = 4–6). Scale bar, 100 µm (×200) and 50 µm (×400). **d** The percentage of IL-17-producing cells from mesenteric lymph nodes of ND, DCA, ND + TNBS and DCA + TNBS treated mice. **e** RORγt (green) and DAPI (blue) immunofluorescence analysis of colon sections from ND, DCA, ND + TNBS and DCA + TNBS treated mice (Scale bar, 50 µm). ***p < 0.001; ****p < 0.0001 compared to the normal diet control mice. ^##^*p* < 0.01 compared to the DCA + TNBS treated mice. One-way ANOVA with Newman-Keuls multiple comparisons test (c) was used. Data are expressed as mean ± SEM from at least three independent experiments or representative data (**b**, **d**, **e**)

Th17 cells have been believed to play a major role in TNBS-induced colitis [34, 35]. To further investigate the role of DCA in the development of colitis, mice were fed with normal chow diet or DCA-supplemented diet respectively, and then TNBS-induced colitis was established in some of the mice in each group (Fig. 7a). Intriguingly, DCA feeding alone was observed to induce certain increment of Th17 cell generation and colonic inflammation, moreover, TNBS-treated mice fed with DCA-supplemented diet caused more severe colitis and tissue damage than those fed with normal chow diet (Fig. 7b–e). Of note, Th17 cells in mesenteric lymph nodes and colonic tissue were both elevated dramatically in DCA-feeding TNBS-treated group (Fig. 7d, e). These results provide further evidence that enhancement of Th17 differentiation may be an important pathogenic mechanism that DCA participates in the HFD-related colonic inflammation.

## Discussion

**Fig. 8:**
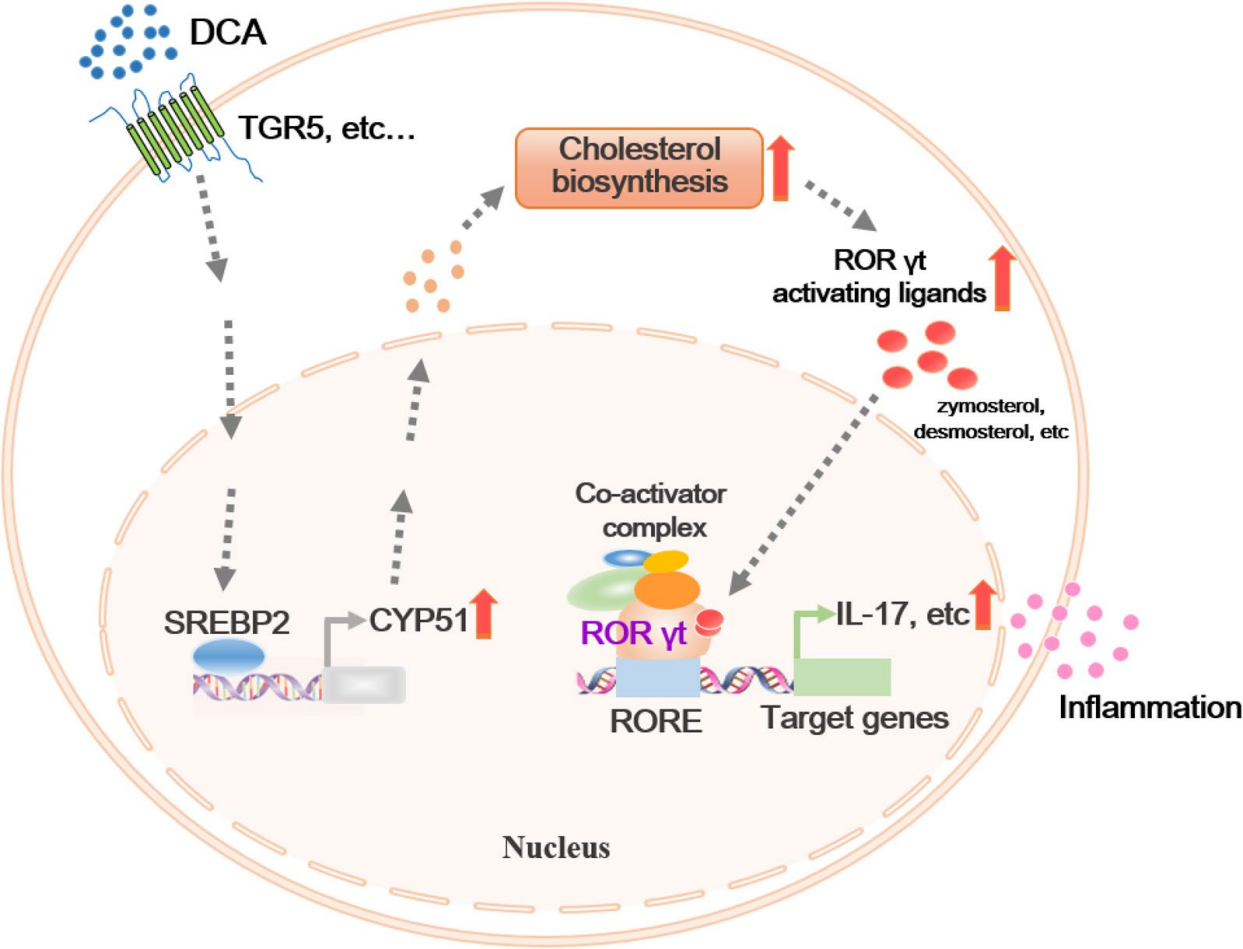
Proposed model of DCA-boosted Th17 differentiation. DCA modulates cholesterol biosynthetic pathway and increases the generation of endogenous RORγt agonists, involving zymosterol and desmosterol, leading to the recruitment of coactivators and transcriptional activation of RORγt target genes, including IL17, therefore promoting inflammation and tissue injury

High-fat diet (HFD) consumption could increase the risk of the occurrence and relapse of IBD. Although high colonic DCA level caused by microbiota alteration play a critical role, detailed mechanisms involved in the DCA-induced intestinal inflammation have still not been clearly elucidated. Our previous data have shown that excess DCA can affect the innate immune system [15, 16]. In the current study, we investigated whether increased DCA level might have a direct effect on CD4^+^ T cell populations. Here we described that DCA could dose-dependently promote in vitro Th17 cell differentiation and pro-inflammatory cytokines production. Importantly, high level DCA modulated cholesterol biosynthetic pathway and significantly up-regulated the expression of CYP51, a key enzyme involved in the process of cholesterol biosynthesis in cells, and inhibition of CYP51 largely abrogated DCA-induced Th17 differentiation. Furthermore, SREBP2 is the major transcription factor responsible for the increment of CYP51 level induced by DCA (Fig. 8). Animal experiments exhibited that high colonic DCA level correlated with in vivo Th17 cell differentiation and inflammatory tissue injury. Therefore, our findings provide a new mechanism that high level DCA-promoted Th17 cell differentiation also participates in the HFD-associated colonic inflammation.

Accumulating evidence has indicated that microbiota exert various effects on host physiology and pathology process especially by producing bioactive metabolites, including trimethylamine *N*-oxide, short-chain fatty acids as well as primary and secondary bile acids [36]. Westernized lifestyle, characterized by long term high fat diet, leads to disturbance of gut microbiota and their metabolites, which serve as central regulators in maintaining intestinal homeostasis by interacting with immune system. As important microbial metabolites affected by diet, the effects of bile acids (BA) on immune response have recently attracted increasing attention [37–40]. Th17 cell, a subset of CD4^+^ helper T cell with prominent pro-inflammatory roles, plays a pivotal part in the pathogenesis of various autoimmune and inflammatory pathologies, including IBD. Dysregulated Th17 differentiation leads to induction and persistence of tissue inflammation through secreting pro-inflammatory cytokines, such as IL-17, and antagonism of Th17 cell associated pathway could greatly ameliorate disease severity [41–43]. Multiple factors have been reported to be implicated in the regulation of Th17 differentiation. CIP2A (Cancerous Inhibitor of Protein Phosphatase 2A) could control Th17 differentiation by down-regulating STAT3 phosphorylation and thus decrease IL-17 production [44]. TACI (transmembrane activator and calcium modulator and cyclophilin ligand interactor) was proved to negatively regulate Th17 function through restricting calcineurin-NFAT signaling axis, accordingly, TACI deficiency could promote Th17 differentiation and result in more severe colitis in mice [45]. In addition, leptin signaling pathway was found to be involved in CD4^+^ T cells differentiating into Th17 lineage, and leptin receptors antagonists could attenuate thyroid inflammation by altering Th17 and Treg proportions [46]. Importantly, it has been reported that HFD-caused obesity selectively promotes the differentiation and proliferation of Th17 cells in vivo*,* moreover, HFD-fed mice develop more severe colitis in TNBS induced model due to the increased Th17 bias [33]. However, what drives the Th17 differentiation under the HFD settings remains largely unclear. DCA is the major secondary bile acid in human colon and exclusively formed via 7α-dehydroxylation by colonic bacteria restricting to the genus *Clostridium* [47]*.* The percentage of *Clostridium* expanded dramatically accompanied by potently increased fecal DCA on HFD conditions [16]. Our in vitro data proved that high level DCA is conductive to promote Th17 cell differentiation with pathogenic properties, meanwhile, in vivo study confirmed that HFD could cause mild colonic inflammation with increased Th17 cell differentiation, and intriguingly, reducing BA level in colon by cholestyramine administration significantly alleviated intestinal inflammation and decreased Th17 cell percentages. Together with our findings showing that TNBS-treated mice fed with DCA-supplemented diet could produce more pronounced intestinal inflammation and much more Th17 cells within mesenteric lymph nodes and colonic tissue than TNBS control counterparts, these results indicate that excessive DCA could be a critical factor contributing to HFD-related Th17 cell bias and colonic inflammation.

Consistent with our findings, high level DCA also contributes to gastric inflammation. DCA is defined as the main bile acid component of duodenogastric reflux, and long-term increment of DCA level leads to abnormal BA metabolism and microbial dysbiosis in the stomach, resulting in gastric inflammation and intestinal meta-plasia development by promoting nuclear STAT3 phos-phorylation and KLF5 upregulation [48]. Nevertheless, in contrast to the accumulating evidence showing that DCA positively correlates with colitis, colorectal and liver cancer, very recent studies disclose its beneficial effects on diseases, such as *Clostridium perfringens* infection and *Campylobacter jejuni* colonization [49, 50]. Wang et al. reported that DCA ameliorated *C. perfringens*-induced chicken necrotic enteritis (NE) by inhibiting cyclooxygenase (COX) signaling pathways and decreasing ileal tissue accumulation of inflammatory mediators [49]. DCA could also prevented *C*. *jejuni* chicken colonization by modulating microbiota composition with increased phylum Bacteroidetes and decreased Firmicutes, suggesting that DCA can exert its effect through interacting with microbiota [50]. Therefore, the role of DCA may be a double-edged sword and why DCA ameliorate infectious enteritis but promote HFD-associated intestinal inflammation need to be further investigated. Considering the multiple role of DCA in diseases, its in vivo level should be tightly controlled. As the most abundant secondary bile acids in colon, DCA is produced through 7α-dehydroxylation reactions from cholic acid (CA). Masanori and colleagues found that a set of six enzymes is necessary and sufficient for the conversion of CA to DCA [51], thereby clarifying the molecular basis for modulating DCA production, which provides new therapeutic targets for corresponding diseases that DCA concentration need to be manipulated.

RORγt is regarded as the key transcription factor controlling the differentiation of naive CD4^+^ T cells into Th17 lineage, and currently it has become an important therapeutic target for Th17 mediated autoimmune diseases and inflammatory disorders [26, 52–54]. As a member of the nuclear hormone receptor super-family, RORγt functions as a ligand-dependent transcriptional factor and holds a highly-conserved DNA binding domain (DBD) at the N terminus, a hinge domain and a ligand binding domain (LBD) at the C terminus. Specific ligands of RORγt can directly bind to its ligand binding domain (LBD), thus inducing the recruitment of co-activators or co-repressors to regulate transcriptional activity of RORγt and subsequently dictate the differentiation of Th17 cells. Over the last several years, a number of cholesterol biosynthetic intermediates (CBIs) have been recognized as endogenous natural ligands of RORγt, thus establishing a link between cholesterol metabolism and Th17 differentiation [27]. The CBIs, especially zymosterol, zymosteron, desmosterol and dehydrocholesterol, have been identified among the most potent sterols activating RORγt [29]. Intriguingly, desmosterol was proved to be essential for Th17 differentiation and it can selectively activating RORγt without obvious effect on the differentiation of Th1 and other Th subgroups [27]. Consistent with these reports, our data showed that high level DCA treatment could significantly increase levels of zymosterol and desmosterol in T cells, which may contribute to the promoting role of DCA on Th17 differentiation. Furthermore, cholesterol biosynthetic process is mediated by a number of enzymes, while CYP51, a kind of cytochrome p450s, is identified as the critical enzyme in the cholesterol biosynthetic pathway responsible for generating RORγt agonist [25]. Disruption of CYP51 expression could reduce RORγt transcriptional activity and Th17 differentiation by decreasing the synthesis of endogenous RORγt agonists, including zymosterol and desmosterol, whereas CYP51 over-expression could significantly increase the transcriptional activity of RORγt. Our results indeed exhibited that the expression of CYP51 was dramatically up-regulated in response to DCA stimulation, and CYP51 inhibition as well as cholesterol synthesis blockage successfully reversed the effect of DCA on Th17 differentiation, therefore highly suggesting that excessive DCA could enhance the availability of RORγt agonists by modulating cellular cholesterol synthetic pathway and consequently promote the differentiation of CD4^+^T cell into Th17 linage.

Additionally, our data showed that sterol regulatory element-binding protein 2 (SREBP 2) is the major transcription factor mediating the considerable up-regulation of CYP51 by DCA. SREBPs are dominant modulators of lipid homeostasis that control the expression of genes involved in lipid cholesterol synthesis. SREBP1 is primarily involved in fatty acid synthesis, while SREBP2 preferentially promotes cellular cholesterol synthesis. Upon activation, N-terminal portion of SREBP2 translocates into the nucleus and binds to the sterol response elements (SREs) activating target genes that encode cholesterol biosynthetic enzymes [55]. Recently, the important role of SREBP2 in inflammation is increasingly appreciated. Paralleled with our findings showing that SREBP2 participated in the orchestration of adaptive immune cells by DCA to elicit inflammatory response, SREBP2 was also found to engage in TNF-induced pro-inflammatory macrophage polarization as well as activate NLRP3 inflammasome in macrophages in the form of SCAP-SREBP2 complex to mediate inflammation [56, 57]. Furth more, SREBP2 could promote NLRC4 inflammasome activation in keratinocytes and thus has been implicated in the LCN2-induced psoriatic inflammation [58]. More recently, SREBP-2 was reported to be highly activated in COVID-19 patients’ PBMCs and contributed to the generation of cytokine storm, while SREBP2 inhibitor could suppress cytokine storm and prevent pulmonary damages in a virus infection mouse model [59]. Together, these findings indicate that SREBP2 could be a promising therapeutic target for inflammatory diseases including HFD-associated meta-inflammation status.

## Limitations of the study

Although our study showed that DCA can facilitate Th17 differentiation by affecting cholesterol biosynthesis pathway and contribute to the colonic inflammation, some limitations should be considered. In vivo data exhibited that DCA-supplemented diet promotes the infiltration of intestinal Th17 cells and aggravates TNBS-induced colitis. This model cannot exclude the role of other possible DCA-responsive immune cells in intestine in the development of colitis, therefore, it would be better to confirm the mechanisms by establishing CD4-specific Rorc deficient mice model in the future studies. Moreover, further analysis with single-cell RNA sequencing would comprehensively map the pattern of intestinal immune cell populations upon DCA administration. In addition, DCA can also affect the composition of gut microbiota, and the consequent contributions to the development of HFD-induced colitis remains to be investigated.

## Conclusions

In summary, our study reveals that HFD-induced high level DCA could act on cholesterol metabolic pathway in CD4^+^ T cells, thereby enlarging the intracellular pool of RORγt agonists and then promoting pro-inflammatory Th17 polarization, which highlights the effect of excessive DCA on adaptive immune system and provides a novel mechanistic insight into how high fat diet drives colonic inflammation. Combined with our previous findings prove that high level DCA could also induce macrophages pro-inflammatory phenotype switch, these results suggest that using bile acid sequestrants as well as targeting the corresponding pathways that DCA is implicated in, for instance, disturbing the cholesterol metabolic-inflammatory crosstalk, may represent plausible strategies for prevention and treatment of IBD and other HFD-related inflammatory disorders.

## Methods

### Reagents

Deoxycholic acid and JTE-013 were obtained from Sigma-Aldrich (St. Louis, MO, USA). Triamterene, 4-DAMP, ketoconazole, lovastatin, KG-501 and fatostatin were purchased from MedChemexpress (Monmouth Junction, NJ, USA). Methoctramine was from AdooQ Bioscience (Nanjing, China). Z-Guggulsterone was from Santa cruz Biotechnology (Santa cruz, CA, USA). SREBP2 antibody was obtained from R&D Systems (Minneapolis, MN, USA).

### Mice

6- to 8-week-old C57BL/6 male mice were purchased from Experimental Animal Center of the Chinese Academy of Sciences (Shanghai, China) and maintained under a humidity-controlled specific pathogen free (SPF) facility. All the animal study protocols complied with the Guide for the Care and Use of Medical Laboratory Animals issued by the Ministry of Health of China and approved by the Ethics Committee of Xinhua Hospital Affiliated to Shanghai Jiaotong University School of Medicine.

### HFD animal experiment

The mice were fed with normal chow diet (ND, 12 kcal% fat) or high-fat diet (HFD, 60 kcal% fat) ad libitum for 4 months. For cholestyramine-treatment group, mice were fed with HFD supplemented with 2% cholestyramine (Sigma-Aldrich, St. Louis, MO, USA). Body weight was monitored weekly during entire course of experiment. Fresh fecal samples were collected for the fecal DCA detection by LC–MS/MS analysis. Mice were sacrificed after 4 months feeding and lymphocytes from mesenteric lymph node (MLN) were harvested for flow-cytometry detection. Colon tissue was fixed in 4% buffered formalin and embedded in paraffin. Then the sections of murine colon were used for HE staining and immunofluorescent analysis.

### TNBS colitis induction and treatment

The mice were randomly divided into four groups, including normal diet group (ND), DCA diet group (normal diet supplemented with 0.2% DCA), ND + TNBS (Sigma-Aldrich, St. Louis, MO, USA) group and DCA diet + TNBS group (n = 7 per group). All the mice in the ND + TNBS and DCA diet + TNBS group were pre-sensitized by application of 1% TNBS (w/v in a 4:1 volume ratio of acetone and olive oil) on the skin at the fifth week, and then underwent rectal enema of 2.5% TNBS (100 μL, 5% TNBS dissolved in absolute ethanol at 1:1 volume ratio) a week later as described previously [60]. 4 days after TNBS treatment, mice were sacrificed and MLNs were collected for flow-cytometry analysis. Colon tissue was prepared as mentioned above.

### Histological analysis

The paraffin sections of colonic tissue were stained with hematoxylin and eosin (H&E) and histopathologic scoring was assessed as described previously in a blinded manner [61], including aberrant crypt architecture (0–5), crypt abscesses (0–3), increased crypt length (0–4), goblet cell loss (0–3), tissue damage (0–3), inflammatory cell infiltration (0–3) and lamina propria neutrophils counts (0–3). The combined score ranged from 0 to 24.

### Cells

EL4 cell line was obtained from the Type Culture Collection of the Chinese Academy of Sciences (Shanghai, China). Cells were cultivated in RPMI1640 culture medium supplemented with 10% fetal bovine serum (Gibico, CA, USA) and 1% penicillin/streptomycin (Invitrogen, CA, USA) at 37 °C with 5% CO_2_. Naive CD4^+^ T cells were isolated and purified from spleens and lymph nodes of wild-type C57BL/6 mice using a CD4 microbead kit (Miltenyi Biotec, Bergisch Gladbach, German). Cells with a purity of > 90% were used for the following experiments.

### CD4^+^ T cell differentiation in vitro

Naive CD4^+^ T cells (1 × 10^5^ per well) were activated with anti-CD3 (Invitrogen, CA, USA) and soluble anti-CD28 (Invitrogen, CA, USA) for 72 h in 96-well microplates in the presence of polarization cytokines and blocking antibodies. Then the cells were restimulated by PMA plus ionomycin together with brefeldin A for 5 h, and intracellular cytokines were detected by flow cytometry. For Th1 differentiation, IL-12 (PeproTech, NJ, USA) and anti-IL-4 (BD Biosciences, CA, USA) were used; For Th2 differentiation, IL-4 (PeproTech, NJ, USA) and anti-IFN-γ (BD Bioscience, CA, USA) were applied; For Th17 differentiation, cells were stimulated with IL-6 (R&D Systems, Minneapolis, MN, USA), TGF-β (R&D Systems, Minneapolis, MN, USA) in the presence of anti-IL-4 and anti-IFN-γ antibodies. For DCA stimulation experiments, various dosages of DCA was added to the culture medium 24 h after CD4^+^ T cells activation and indicated inhibitors were added 30 min ahead of DCA treatment.

### Flow cytometry

Cells were stained with anti-CD4-APC (Invitrogen, CA, USA) or anti-CD4-PE (Invitrogen, CA, USA) antibody for 30 min at 4˚C. Next, cells were fixed with IC Fixation Buffer followed by incubation with permeabilization buffer, and stained with anti-IL-17-PE (BD Pharmingen, CA, USA), anti-IFN-γ-APC (Invitrogen, CA, USA) or anti-IL-4-PE (Invitrogen, CA, USA) antibodies. Data were acquired on a FACSCanto II flow cytometer (BD Biosciences, USA) and analyzed using FlowJo software (Tree Star, Inc., Ashland, OR, USA).

### Real-time PCR

Total RNA was purified using an RNeasy Mini kit (Qiagen, German) and then was reverse transcribed into cDNA by PrimeScript RT Master Mix (Takara, Japan). The mRNA expression levels of CYP51, IL-17A, IL-17F, IL-21, IL-22, GM-SF, CCR6, IL23R and GZMB were determined by Real-time PCR on the PikoReal Real-Time PCR System (Thermo, USA) using primers: Cyp51, forward 5'-GAC AGG AGG CAA CTT GCT TTC-3', reverse 5'-GTG GAC TTT TCG CTC CAG C-3'; IL-17A, forward 5'-CTC CAG AAG GCC CTC AGA CTA C-3', reverse 5'-AGC TTT CCC TCC GCA TTG ACA CAG-3'; IL-17F, forward 5'-AGG AAG ACA GCA CCA TGA ACT-3', reverse 5'-GAG CAT CTT CTC CAA CCT GAA-3'; IL-21, forward 5'-CGC CTC CTG ATT AGA CTT CG-3', reverse 5'-GCC CCT TTA CAT CTT GTG GA-3'; IL-22, forward 5'-CAT GCA GGA GGT GGT ACC TT-3', reverse 5'-CAG ACG CAA GCA TTT CTC AG-3'; GM-CSF, forward 5'-CCG TAG ACC CTG CTC GAA TA-3', reverse 5'-TGC CTG TCA CAT TGA ATG AA-3'; CCR6, forward 5'-GTC CAA GTT CAA CCA GCA CC-3', reverse 5'-GTT GGT GAG CTT TAG CTT CC-3'; GZMB, forward 5'-CCA CTC TCG ACC CTA CAT GG-3', reverse 5'-GGC CCC CAA AGT GAC ATT TAT T-3'.

### Western blot

EL4 cells were incubated without or with different dosage of DCA for 24 h, and the cells were collected and lysed in protein lysis buffer containing protease and phosphatase inhibitors (Theromo Fisher Scientific), then cell lysates were resolved by SDS-PAGE, transferred to PVDF membranes (0.2 μm) and probed with antibodies against CYP51 (#D227358, Sangon Biotech, China). Membranes were then incubated with a stripping buffer (Thermo Fisher Scientific) for 30 min and re-probed with antibodies against β-actin (#A1978, sigma, USA). Reactive signals were detected by ECL Western Blotting Substrate (Thermo Fisher Scientific) and quantified by ChemiDoc™ XRS^+^ System (Bio-Rad).

### RNA-seq analysis

Primed CD4^+^ T cells were treated with or without DCA (100 μM) for 4 h. Total RNA was isolated using the RNeasy kit (Qiagen, German) according to the manufacturer’s instructions. RNA-seq libraries were subjected to QC analysis and sequenced on DNBSEQ platform. Clean reads were mapped to the reference genome using HISAT2 (v2.0.4), and Bowtie2 (v2.2.5) was applied to align the clean reads to the reference coding gene set, then gene expression level was calculated by RSEM (v1.2.12). Differential gene expression analysis was performed using DESeq2 (v1.4.5). GO and KEGG enrichment analysis was implemented by Phyper based on Hypergeometric test. Statistical significance was corrected by Q value with a rigorous threshold (Q value ≤ 0.05) by Bonferroni. All the raw data were available at SRA (https://www.ncbi.nlm.nih.gov/sra) with accession code PRJNA898494.

### Cholesterol metabolism pathway analysis by LC–MS

To analyze sterol lipids metabolism, lipids were extracted from EL4 cells untreated or treated with DCA using a modified version of Bligh and Dyer’s protocol [62]. 500 μL of ethanol containing 5 µg of BHT and an internal standard cocktail (50 µL) were added into the lipid extracts mentioned above. Samples were incubated for 15 min at 1200 rpm (4 ℃), and 250 µL of Milli-Q water together with 1 ml of *n*-hexane were added. Samples were then mixed by vortexing and centrifuged at 12,000 rpm for 5 min (4 ℃). Clear upper phase containing sterols in hexane was transferred to a new tube. Extraction was repeated once with another 1 ml of *n*-hexane and pooled extracts were dried in a SpeedVac under organic mode. Sterols were derivatized to obtain their picolinic acid esters and then analyzed on a Thermofisher DGLC U3000 coupled with Sciex QTRAP 6500 Plus, and subsequently quantitated by referencing the spiked internal standards as previously described [63]. The concentrations of sterol lipids in each group were standardized to Z-scores.

### Chromatin immunoprecipitation assay

ChIP assay was performed with a kit (Beyotime, Shanghai, China) according to the manufacturer’s protocols. EL4 cells were treated as indicated and cross-linked by 1% formaldehyde. Nuclei were collected and subjected to sonication. Then the chromatin extracts were pre-cleared with protein A + G agarose and immunoprecipitated with SREBP2 antibody (#AF7119, R&D Systems, MN, USA) or rabbit IgG (#AC005, ABclonal, Wuhan, China) overnight (4 °C). De-crosslinking was implemented followed by DNA purification. Finally, PCR amplification was performed using primers (forward: 5′-GAA GGG CTG GTC TCA CAA AG-3′, and reverse: 5′-CGA AGG CGC TCT GTG ATT G-3′) encompassing the predicted SREBP2 binding region of murine *Cyp51* promoter.

### Immunofluorescence staining

EL4 cells were untreated or treated with DCA (100 μM) and fixed with 4% paraformaldehyde (PFA) for 30 min. Cells were then permeabilized with 0.1% Triton X-100 (10 min) followed by incubation with SREBF2 primary antibody (1:25, 90 min) at room temperature. Donkey Anti-Goat IgG (H + L) -PE was used as secondary antibody (1:200, 30 min). Nuclei were then stained with DAPI for 5 min and SREBP2 localization was visualized under a fluorescence microscope (Leica).

### Statistics

Data were expressed as mean ± standard error of the mean (s.e.m.). For 2-group comparison, Shapiro–Wilk test and F test were used to evaluate the normal distribution and homogeneity of variance of data respectively. Shapiro–Wilk test was satisfactory in all cases, and then statistical significance was assessed by two-tailed Student’s *t*-test or *t*-test with Welch's correction. One-way analysis of variance (ANOVA) with Tukey's multiple comparisons test or Newman-Keuls multiple comparisons test was used for multiple-group comparisons. Differences were considered to be statistically significant for *p* < 0.05.

### Supplementary Information

**Additional file 1: Figure S1.** Heat map depicting relative mRNA expression of cholesterol metabolism related genes in untreated and DCA-treated CD4^+^T cells, related to Fig. 3. **Figure S2.** Western-blot analysis of CYP51 protein expression upon DCA stimulation (uncropped gels), related to Fig. 3.

## Data Availability

The data of current study will be available from the corresponding author on reasonable request.

## Abbreviations

HFD: High-fat diet
IBD: Inflammatory bowel disease
DCA: Deoxycholic acid
DAMP: Damage-associated molecular pattern
TLR2: Toll-like receptor 2
CYP51: Lanosterol 14alpha-demethylase
RORγt: Retinoic acid receptor-related orphan receptor γt
S1PR2: Sphingosine-1-phosphate receptor 2
M2-mAchR: M2 muscarinic acetylcholine receptor
FXR: Farnesoid X-receptor
CREB: CAMP responsive element binding protein
SREBP: Sterol-regulatory element binding protein
CBI: Cholesterol biosynthetic intermediate
SRE: Sterol response element

## References

[1] Hou JK, Abraham B, El-Serag H (2011). Dietary intake and risk of developing inflammatory bowel disease: a systematic review of the literature. Am J Gastroenterol.

[2] Li T, Qiu Y, Yang HS, Zhuang XJ, Zhang SH, Feng R (2020). Systematic review and meta-analysis: association of a pre-illness Western dietary pattern with the risk of developing inflammatory bowel disease. J Dig Dis.

[3] Nabhani ZA, Dulauroy S, Lécuyer E, Polomack B, Campagne P, Berard M (2019). Excess calorie intake early in life increases susceptibility to colitis in adulthood. Nat Metab.

[4] Richman E, Rhodes JM (2013). Evidence-based dietary advice for patients with inflammatory bowel disease. Aliment Pharmacol Ther.

[5] Duan Y, Zeng L, Zheng C, Song B, Li F, Kong X (2018). Inflammatory links between high fat diets and diseases. Front Immunol.

[6] Antonioli L, Caputi V, Fornai M, Pellegrini C, Gentile D, Giron MC (2019). Interplay between colonic inflammation and tachykininergic pathways in the onset of colonic dysmotility in a mouse model of diet-induced obesity. Int J Obes (Lond).

[7] Kawano Y, Nakae J, Watanabe N, Kikuchi T, Tateya S, Tamori Y (2016). Colonic pro-inflammatory macrophages cause insulin resistance in an intestinal Ccl2/Ccr2-dependent manner. Cell Metab.

[8] Stenman LK, Holma R, Forsgard R, Gylling H, Korpela R (2013). Higher fecal bile acid hydrophobicity is associated with exacerbation of dextran sodium sulfate colitis in mice. J Nutr.

[9] Liu TC, Kern JT, Jain U, Sonnek NM, Xiong S, Simpsonet KF (2021). Western diet induces Paneth cell defects through microbiome alterations and farnesoid X receptor and type I interferon activation. Cell Host Microbe.

[10] Fu T, Coulter S, Yoshihara E, Oh TG, Fang S, Cayabyab F (2019). FXR regulates intestinal cancer stem cell proliferation. Cell.

[11] Yoshimoto S, Loo TM, Atarashi K, Kanda H, Sato S, Oyadomari S (2013). Obesity-induced gut microbial metabolite promotes liver cancer through senescence secretome. Nature.

[12] Stenman LK, Holma R, Korpela R (2012). High-fat-induced intestinal permeability dysfunction associated with altered fecal bile acids. World J Gastroenterol.

[13] Traub RJ, Tang B, Ji Y, Pandya S, Yfantis H, Sun Y (2008). A rat model of chronic postinflammatory visceral pain induced by deoxycholic acid. Gastroenterology.

[14] Bernstein H, Holubec H, Bernstein C, Ignatenko N, Gerner E, Dvorak K (2006). Unique dietary-related mouse model of colitis. Inflamm Bowel Dis.

[15] Zhao S, Gong Z, Zhou J, Tian C, Gao Y, Xu C (2016). Deoxycholic acid triggers NLRP3 inflammasome activation and aggravates DSS-induced colitis in mice. Front Immunol.

[16] Wang L, Gong Z, Zhang X, Zhu F, Liu Y, Jin C (2020). Gut microbial bile acid metabolite skews macrophage polarization and contributes to high-fat diet-induced colonic inflammation. Gut Microbes.

[17] Geremia A, Biancheri P, Allan P, Corazza GR, Sabatino AD (2014). Innate and adaptive immunity in inflammatory bowel disease. Autoimmun Rev.

[18] Silva FA, Rodrigues BL, Ayrizono ML, Leal RF (2016). The immunological basis of inflammatory Bowel disease. Gastroenterol Res Pract.

[19] Iwakura Y, Ishigame H, Saijo S, Nakae S (2011). Functional specialization of interleukin-17 family members. Immunity.

[20] Yen D, Cheung J, Scheerens H, Poulet F, McClanahan T, McKenzie B (2006). IL-23 is essential for T cell mediated colitis and promotes inflammation via IL-17 and IL-6. J Clin Invest.

[21] Jiang W, Su J, Zhang X, Cheng X, Zhou J, Shi R (2014). Elevated levels of Th17 cells and Th17-related cytokines are associated with disease activity in patients with inflammatory bowel disease. Inflamm Res.

[22] Paulissen SM, Hamburg JP, Dankers W, Lubberts E (2015). The role and modulation of CCR6^+^ Th17 cell populations in rheumatoid arthritis. Cytokine.

[23] Hou L, Yuki K (2022). CCR6 and CXCR6 identify the Th17 cells with cytotoxicity in experimental autoimmune encephalomyelitis. Front Immunol.

[24] Codarri L, Gyülvészi G, Tosevski V, Hesske L, Fontana A, Magnenat L (2011). ROR gammat drives production of the cytokine GM-CSF in helper T cells, which is essential for the effector phase of autoimmune neuroinflammation. Nat Immunol.

[25] Santori FR, Huang P, Pavert SA, Douglass-Jr EF, Leaver DJ, Haubrich BA (2015). Identification of natural RORγ ligands that regulate the development of lymphoid cells. Cell Metab.

[26] Ivanov II, McKenzie BS, Zhou L, Tadokoro CE, Lepelley A, Lafaille JJ (2006). The orphan nuclear receptor ROR gammat directs the differentiation program of proinflammatory IL-17^+^ T helper cells. Cell.

[27] Hu X, Wang Y, Hao LY, Liu X, Lesch CA, Sanchez B (2015). Sterol metabolism controls T(H)17 differentiation by generating endogenous RORg agonists. Nat Chem Biol.

[28] Soroosh P, Wu J, Xue X, Song J, Sutton SW, Sablad M (2014). Oxysterols are agonist ligands of RORγt and drive Th17 cell differentiation. Proc Natl Acad Sci USA.

[29] Jetten AM, Takeda Y, Slominski A, Kang HS (2018). Retinoic acid-related Orphan Receptor γ (RORγ): connecting sterol metabolism to regulation of the immune system and autoimmune disease. Curr Opin Toxicol.

[30] Schaap FG, Trauner M, Jansen PLM (2014). Bile acid receptors as targets for drug development. Nat Rev Gastroenterol Hepatol.

[31] Zhou H, Hylemon PB (2014). Bile acids are nutrient signaling hormones. Steroids.

[32] Halder SK, Fink M, Waterman MR, Rozman D (2002). A cAMP-responsive element binding site is essential for sterol regulation of the human lanosterol 14alpha-demethylase gene (CYP51). Mol Endocrinol.

[33] Winer S, Paltser G, Chan Y, Tsui H, Engleman E, Winer D (2009). Obesity predisposes to Th17 bias. Eur J Immunol.

[34] Jin Yu, Lin Y, Lin L, Zheng C (2012). IL-17/IFN-γ interactions regulate intestinal inflammation in TNBS-induced acute colitis. J Interferon Cytokine Res.

[35] He C, Shi Y, Ruijin Wu, Sun M, Fang L, Wei Wu (2016). miR-301a promotes intestinal mucosal inflammation through induction of IL-17A and TNF-α in IBD. Gut.

[36] Gambardella J, Castellanos V, Santulli G (2021). Standardizing translational microbiome studies and metagenomic analyses. Cardiovasc Res.

[37] Cao W, Kayama H, Chen ML, Delmas A, Sun A, Kim SY (2017). The xenobiotic transporter Mdr1 enforces T cell homeostasis in the presence of intestinal bile acids. Immunity.

[38] Hang S, Paik D, Yao L, Kim E, Trinath J, Lu J (2019). Bile acid metabolites control TH17 and Treg cell differentiation. Nature.

[39] Campbell C, McKenney PT, Konstantinovsky D, Isaeva OI, Schizas M, Verter J (2020). Bacterial metabolism of bile acids promotes peripheral Treg cell generation. Nature.

[40] Li W, Hang S, Fang Y, Bae S, Zhang Y, Zhang M (2021). A bacterial bile acid metabolite modulates Treg activity through the nuclear hormone receptor NR4A1. Cell Host Microbe.

[41] Liu ZJ, Yadav PK, Su JL, Wang JS, Fei K (2009). Potential role of Th17 cells in the pathogenesis of inflammatory bowel disease. World J Gastroenterol.

[42] Sarra M, Pallone F, Macdonald TT, Monteleone G (2010). IL-23/IL-17 axis in IBD. Inflamm Bowel Dis.

[43] Elson CO, Cong Y, Weaver CT, Schoeb TR, McClanahan TK, Fick RB (2007). Monoclonal anti-interleukin 23 reverses active colitis in a T cell-mediated model in mice. Gastroenterology.

[44] Khan MM, Ullah U, Khan MH, Kong L, Moulder R, Välikangas T (2020). CIP2A constrains Th17 differentiation by modulating STAT3 signaling. iScience..

[45] Tan AH, Tso GHW, Zhang B, Teo PY, Ou X, Ng SW (2020). TACI constrains TH17 pathogenicity and protects against gut inflammation. iScience..

[46] Wang W, Zhang BT, Jiang QL, Zhao HQ, Xu Q, Zeng Y (2022). Leptin receptor antagonist attenuates experimental autoimmune thyroiditis in mice by regulating Treg/Th17 cell differentiation. Front Endocrinol (Lausanne).

[47] Ridlon JM, Kang DJ, Hylemon PB (2006). Bile salt biotransformations by human intestinal bacteria. J Lipid Res.

[48] Jin D, Huang K, Xu M, Hua H, Ye F, Yan J (2022). Deoxycholic acid induces gastric intestinal metaplasia by activating STAT3 signaling and disturbing gastric bile acids metabolism and microbiota. Gut Microbes..

[49] Wang H, Latorre JD, Bansal M, Abraha M, Al-Rubaye B, Tellez-Isaias G (2019). Microbial metabolite deoxycholic acid controls *Clostridium perfringens*-induced chicken necrotic enteritis through attenuating inflammatory cyclooxygenase signaling. Sci Rep.

[50] Alrubaye B, Abraha M, Almansour A, Bansal M, Wang H, Kwon YM (2019). Microbial metabolite deoxycholic acid shapes microbiota against *Campylobacter jejuni* chicken colonization. PLoS ONE.

[51] Funabashi M, Grove TL, Wang M, Varma Y, McFadden ME, Brown LC (2020). A metabolic pathway for bile acid dehydroxylation by the gut microbiome. Nature.

[52] Kojetin DJ, Burris TP (2014). REV-ERB and ROR nuclear receptors as drug targets. Nat Rev Drug Discov.

[53] Wit J, Al-Mossawi MH, Huhn MH, Arancibia-Cárcamo CV, Doig K, Kendrick B (2016). RORγt inhibitors suppress TH17 responses in inflammatory arthritis and inflammatory bowel disease. J Allergy Clin Immunol.

[54] Bassolas-Molina H, Raymond E, Labadia M, Wahle J, Ferrer-Picón E, Panzenbeck M (2018). An RORγt oral inhibitor modulates IL-17 responses in peripheral blood and intestinal mucosa of Crohn’s disease patients. Front Immunol.

[55] Brown MS, Goldstein JL (1997). The SREBP pathway: regulation of cholesterol metabolism by proteolysis of a membrane-bound transcription factor. Cell.

[56] Kusnadi A, Park SH, Yuan R, Pannellini T, Giannopoulou E, Oliver D (2019). The cytokine TNF promotes transcription factor SREBP activity and binding to inflammatory genes to activate macrophages and limit tissue repair. Immunity.

[57] Guo C, Chi Z, Jiang D, Xu T, Yu W, Wang Z (2018). Cholesterol homeostatic regulator SCAP-SREBP2 integrates NLRP3 inflammasome activation and cholesterol biosynthetic signaling in macrophages. Immunity.

[58] Ma J, Chen J, Xue K, Yu C, Dang E, Qiao H (2022). lcn2 mediates skin inflammation in psoriasis through the SREBP2-NLRC4 axis. J Invest Dermatol.

[59] Lee W, Ahn JH, Park HO, Kim HN, Kim H, Yoo Y (2020). COVID-19-activated SREBP2 disturbs cholesterol biosynthesis and leads to cytokine storm. Signal Transduct Target Ther.

[60] Wirtz S, Popp V, Kindermann M, Gerlach K, Weigmann B, Fichtner-Feigl S (2017). Chemically induced mouse models of acute and chronic intestinal inflammation. Nat Protoc.

[61] Heazlewood CK, Cook MC, Eri R, Price GR, Tauro SB, Taupin D (2008). Aberrant mucin assembly in mice causes endoplasmic reticulum stress and spontaneous inflammation resembling ulcerative colitis. PLoS Med.

[62] Lam SM, Zhang C, Wang Z, Ni Z, Zhang S, Yang S (2021). A multi-omics investigation of the composition and function of extracellular vesicles along the temporal trajectory of COVID-19. Nat Metab.

[63] Chen YY, Ge JY, Zhu SY, Shao ZM, Yu KD (2022). Copy number amplification of ENSA promotes the progression of triple-negative breast cancer via cholesterol biosynthesis. Nat Commun.

