# Supplementary material for: Gut microbial metabolite deoxycholic acid facilitates Th17 differentiation through modulating cholesterol biosynthesis and participates in high-fat diet-associated colonic inflammation
Source: Cell Biosci. 2023 Oct 3;13:186. doi: 10.1186/s13578-023-01109-0 (PMC10548658; doi:10.1186/s13578-023-01109-0)
Supplement: Supplementary file 1 — Additional file 1: Figure S1. Heat map depicting relative mRNA expression of cholesterol metabolism related genes in untreated and DCA-treated CD4+T cells, related to Fig. 3. Figure S2. Western-blot analysis of CYP51 protein expression upon DCA stimulation (uncropped gels), related to Fig. 3. [file 13578_2023_1109_MOESM1_ESM.doc]

**Additional file**

**Gut Microbial Metabolite Deoxycholic Acid Facilitates Th17 Differentiation through Modulating Cholesterol Biosynthesis and Participates in High-Fat Diet-Associated Colonic Inflammation**

**Dan Li, Jiefei Zhou, Lingyu Wang, Zizhen Gong, Huijuan Le, Ye Huang, Congfeng Xu, Chunyan Tian, Wei Cai, Jin Wu**

^*^**Address****Correspondence to**: Jin Wu, Phone: +86-21-25076443, Fax: +86-21-65791316, E-mail: wujin@xinhuamed.com.cn; Wei Cai, E-mail: caiw204@sjtu.edu.cn or Chunyan Tian, E-mail: tianchunyan@ncpsb. org.cn

**Inventory of Supplementary Data**

Supplementary Figures and Legends

- - - Figure S1. Heat map depicting relative mRNA expression of cholesterol metabolism related genes in untreated and DCA-treated CD4^+^T cells, related to Figure 3
    - Figure S2. Western-blot analysis of CYP51 protein expression upon DCA stimulation (uncropped gels), related to Figure 3

**Supplementary Figures and Legends**

**
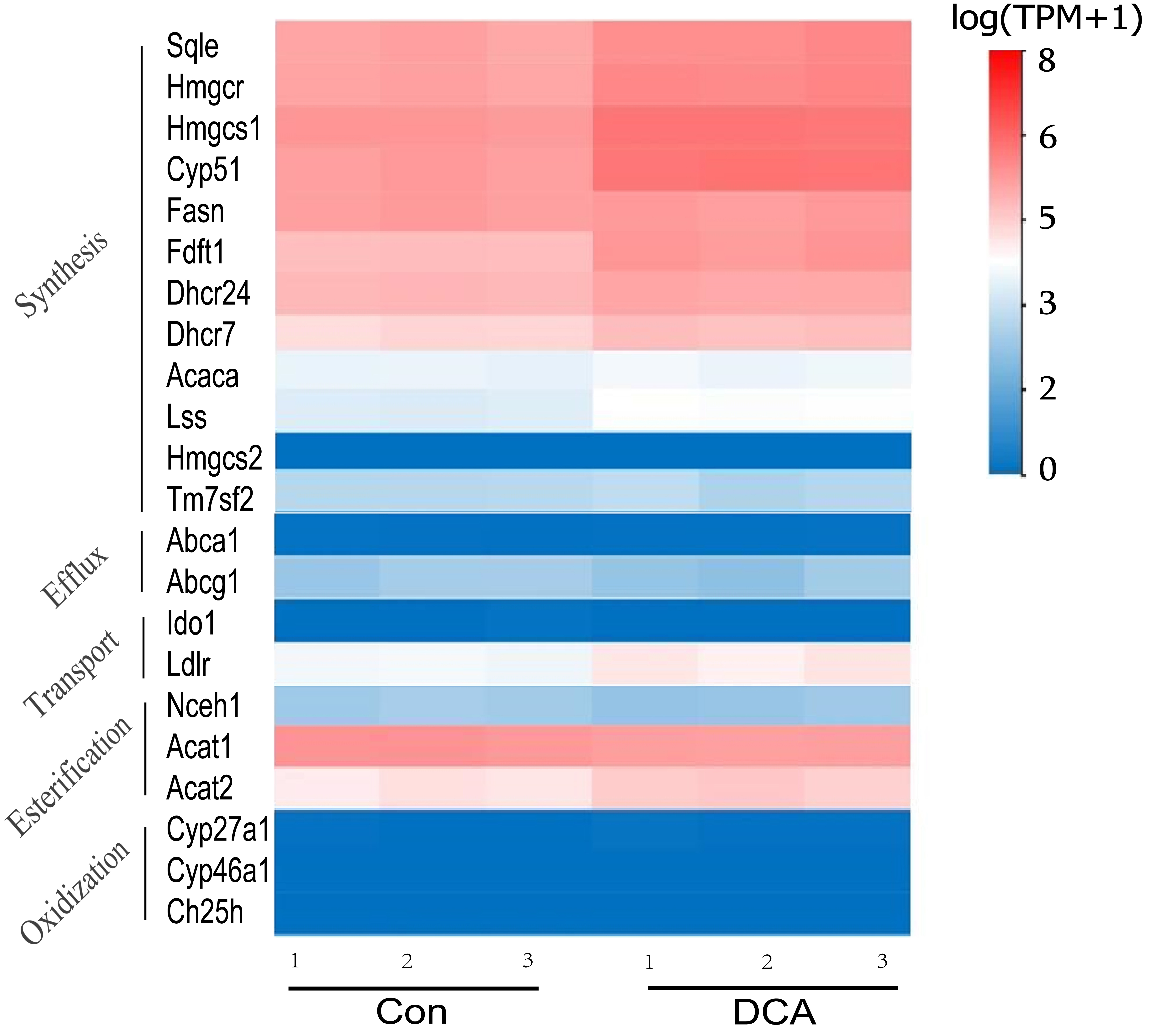
**

**Fig. S1** Heat map depicting relative mRNA expression of cholesterol metabolism related genes in untreated and DCA-treated CD4^+^T cells.

**
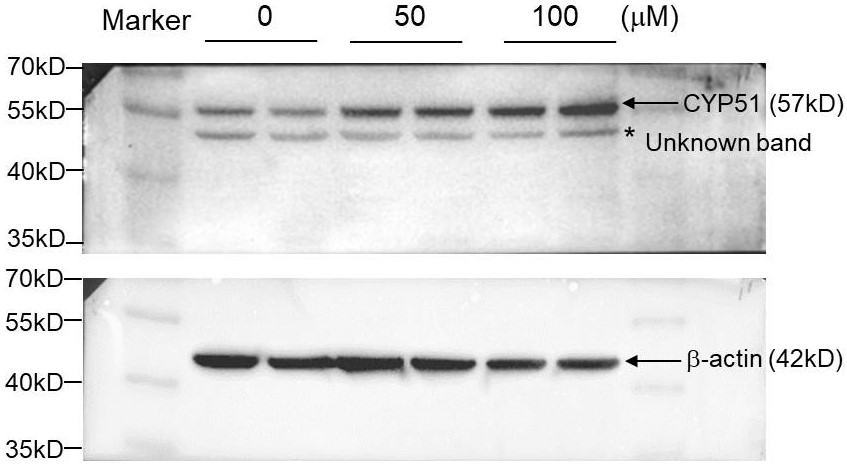
**

**Fig. S2** Western-blot analysis of CYP51 protein expression upon DCA stimulation (original gels). EL4 cells were treated with different dosages of DCA (0, 50, 100 μM)) for 24h. The protein expression level of CYP51 was determined by western blot. β-actin was deemed as a loading control.
